# The Active Recovery Triad Model: A New Approach in Dutch Long-Term Mental Health Care

**DOI:** 10.3389/fpsyt.2020.592228

**Published:** 2020-11-05

**Authors:** Lieke J. C. Zomer, Yolande Voskes, Jaap van Weeghel, Guy A. M. Widdershoven, Tom F. M. M. van Mierlo, Bram S. Berkvens, Bert Stavenuiter, Lisette van der Meer

**Affiliations:** ^1^Department of Ethics, Law and Humanities, Amsterdam UMC, Amsterdam, Netherlands; ^2^Tranzo Scientific Center for Care and Wellbeing, Tilburg School of Social and Behavioral Sciences, Tilburg University, Tilburg, Netherlands; ^3^Impact Care Group, GGz Breburg, Tilburg, Netherlands; ^4^Phrenos Center of Expertise on Severe Mental Illness, Utrecht, Netherlands; ^5^Parnassia Mental Health Center, The Hague, Netherlands; ^6^Reinier van Arkel, 's-Hertogenbosch, Netherlands; ^7^Ypsilon, The Hague, Netherlands; ^8^Department of Clinical & Developmental Neuropsychology, University of Groningen, Groningen, Netherlands; ^9^Department of Rehabilitation, Lentis Psychiatric Institute, Zuidlaren, Netherlands

**Keywords:** long-term mental health care, recovery, care model, development, serious mental illness (SMI)

## Abstract

Unlike developments in short-term clinical and community care, the recovery movement has not yet gained foothold in long-term mental health services. In the Netherlands, approximately 21,000 people are dependent on long-term mental health care and support. To date, these people have benefited little from recovery-oriented care, rather traditional problem-oriented care has remained the dominant approach. Based on the view that recovery is within reach, also for people with complex needs, a new care model for long-term mental health care was developed, the active recovery triad (ART) model. In a period of 2.5 years, several meetings with a large group of stakeholders in the field of Dutch long-term mental health care took place in order to develop the ART model. Stakeholders involved in the development process were mental health workers, policy advisors, managers, directors, researchers, peer workers, and family representatives. The ART model combines an *active* role for professionals, service users, and significant others, with focus on *recovery* and cooperation between service users, family, and professionals in the *triad*. The principles of ART are translated into seven crucial steps in care and a model fidelity scale in order to provide practical guidelines for teams implementing the ART model in practice. The ART model provides guidance for tailored recovery-oriented care and support to this “low-volume high-need” group of service users in long-term mental health care, aiming to alter their perspective and take steps in the recovery process. Further research should investigate the effects of the ART model on quality of care, recovery, and autonomy of service users and cooperation in the triad.

## Introduction

Internationally, the concept “*Recovery*” has gained increased attention in mental health care (1–4). Particularly for people with severe mental illness (SMI), recovery has become an important issue and is acknowledged in (inter)national policy (5–10). SMI is associated with large social and functional impairments as a result of mental illness (e.g., schizophrenia, bipolar disorder, and personality disorder), persistent for a long period of time (>2 years), and requiring coordinated psychiatric care (11, 12). People with SMI often show complex problems at multiple life domains, which makes recovery a difficult concept for them. Full clinical recovery, sustained remission of symptoms, may not be within reach (3). However, recovery defined as a personal process in which persons discover how to live a meaningful and satisfying life despite the limitations of the illness (13), suggests that the process of recovery may be possible regardless of symptoms and social and functional limitations. In this light, recovery can imply (small) steps toward more community participation, and empowerment, enabling people to regain grip on daily life and finding hope and confidence in the possibility of a meaningful and satisfying life.

The recovery movement has had an impact in the Dutch context and has urged the Dutch mental health system to change. To date, these changes mainly affected acute clinical and community care and lead to the development of care models such as Flexible Assertive Community Treatment (FACT), High and Intensive Care (HIC), and Intensive Home Treatment (IHT) (14–16). Despite these necessary developments in short-term clinical and in community care, the recovery movement has not yet gained foothold in services for long-term mental health care (17). This type of care is typically provided for individuals with complex needs for whom living within the community is deemed unlikely (18). For this group of people, models of outpatient care such as FACT or ACT seem to be insufficient. Though, internationally, the setting in which support is provided to this group of service users varies to some extent, people often live in residential psychiatric facilities because of their dependence upon intensive psychiatric care and support. These long-term psychiatric residential care facilities are characterized by a variety of mental health facilities, ranging from long-stay clinical wards to supported or sheltered living accommodations, situated in an institutional setting or in the community.

Following the example of many other European countries, deinstitutionalization has become an important notion in Dutch mental health care. Large mental health organizations reduced the number of beds, decentralized care, and cooperated with community-based services to meet the needs of their service users. Nevertheless, for a small group of service users with complex mental health needs, these intensive residential services remain warranted (18). Estimations on the magnitude of this group are scarce and vary between 10 and 20% of all people with SMI (19–21). In the Netherlands, the group of people who are dependent on 24-h long-term mental health care and support has recently been estimated at 21,000 people (i.e., 10% of people diagnosed with a severe mental illness), of which more than 5,000 people are admitted at long-stay wards or housing facilities in institutional setting, and approximately 16,000 people are living in sheltered accommodations (22, 23). This group of people has largely been neglected, traditional care approaches remained dominant, and health care models that incorporate a recovery-oriented approach are generally focused upon people who (with more of less support) are able to live in the community.

Most people who have received care in an institutional setting for years, live an isolated life, have little family contacts, and no perspective to move to a more independent situation (1). Common daily activities, such as doing groceries, cooking, laundry, but also small working activities, are frequently disrupted, managed by care workers, or performed in an institutional setting (10, 24). Living in an institution has a negative effect on service users, described already in 1976 by Barton as “institutional neurosis” (25). After a long history of crises, admissions, disappointments, and failed attempts to live more independently, service users perceive an institutional setting as a safe environment and accept the status quo. The fear for relapse and readmission also prevails among family members, who want their relative to be in a safe and stable environment. Taking steps toward recovery and more independency equals uncertainty and is often perceived as stressful. After having lived in long-term mental health facilities for decades, people do not believe that life outside a facility is possible (20).

Internationally, concerns about the quality of care in long-term mental health facilities have led to the development of an instrument to assess quality of care within long-term mental health facilities, namely, the Quality Indicator for Rehabilitative Care (QuIRC) (26). Studies using this instrument showed that a higher quality of care was associated with service users' autonomy and their experiences of care (27). Various interventions and rehabilitation approaches designed for people with complex and persistent mental health needs have been described in the literature. Examples are cognitive adaptation training [CAT; (28, 29)], individual placement and support [IPS; (30)], the Boston University approach to Psychiatric Rehabilitation (31), and wellness recovery action plan [WRAP; (32)]. In addition, research showed that focus on an expected maximum length of stay encourages people in their recovery and supports long-stay service users to move to a more independent setting (33).

Even though deinstitutionalization started relatively late in the Netherlands when compared to other countries, recovery-oriented interventions already gained some foothold in Dutch long-term mental health care (34, 35). However, until now, most recovery-oriented interventions and attempts to improve the quality of long-term mental health care focused on separate aspects of care instead of an integral approach to initiate a radical change in this sector. In line with the developments described in the literature and experienced in practice, various stakeholders in the Netherlands took the initiative to change the current approach in long-term mental health care. In an iterative process, mental health care professionals, service users, family members, policy makers, researchers, and other stakeholders from over 15 mental health care organizations in the Netherlands and patient and family associations collaborated to develop a new integral care model: the Active Recovery Triad (ART) model. This article presents the key characteristics of the ART model.

## Materials and Methods

The development of the ART model was an iterative process involving a number of steps. Table 1 presents the steps of this development process.

**Table 1:** The development process of the ART model.

**Step**	**Activity**	**Date**	**Purpose**	**Involved people**
1	First invitational meeting (1 day)	January 2014	Examine the need for change among stakeholders and formation of expert group	101 people, for example, mental health workers, policy advisors, managers, directors, researchers, peer workers, and family representatives
2	Meeting with expert group (2 days)	June 2014	Develop first outline of the model (vision and mission, target population, core elements, title, formation of writing team)	Expert group; 31 people of 14 different mental health care organizations, branch organization of service users and family organizations, and researchers
3	Writing team draws first outline of handbook	June 2014 to September 2014	Work out key characteristics and the vision and mission of the model into a handbook for professionals	Writing team, consisting of six people from the expert group (TvM, LvdM, YV, BB, BS, and JvW)
4	Second invitational meeting (1 day)	September 2014	Reflect upon the outline of the care model	67 people, for example, mental health workers, policy advisors, managers, directors, researchers, peer workers, and family representatives
5	Meeting with expert group (2 days)	December 2014	Investigate the perspectives of service users and family with regard to the ART model and secure these perspectives in the model development process	First session (morning) with five service user representatives or peer workers, five family representatives and four mental health professionals from the expert group. Second session with the total expert group (as described in step 2)
6	Writing team drafts first chapters of the ART handbook	December 2014 to April 2015	Draft first chapters of the ART handbook	Writing team (as described in step 3)
7	Third invitational meeting (1 day)	April 2015	Discuss the content of the handbook and exchange best practices	81 people, for example, mental health workers, policy advisors, managers, directors, researchers, peer workers, and family representatives
8	Writing team develops draft of ART handbook	April 2015 to November 2015	Work out the ART handbook in total	Writing team (as described in step 3)
9	Fourth invitational meeting (1 day)	November 2015	Collect feedback on the ART handbook, in order to finalize it	71 people, for example, mental health workers, policy advisors, managers, directors, researchers, peer workers, and family representatives
10	Publication of ART handbook	June 2016		

First, an invitational meeting (January, 2014) was organized, to examine the need for change among stakeholders. One hundred and one people attended this meeting, including mental health workers, policy advisors, managers, directors, researchers, peer workers, and family representatives, all affiliated with the long-term mental health care. In the meeting, it was concluded that a recovery-oriented model of care was necessary for service users in need of long-term mental health care. Workshops were organized regarding subjects considered important for long-term mental health care (e.g., recovery, self-management, employment, involvement of family, intensive care, and lifestyle). A report describing a national plan of action aimed to improve care for people with severe mental illness was used as source of inspiration (10). At the end of the meeting, an expert group was formed to develop the first outline of the model.

The expert group consisted of 31 people from 14 different mental health care organizations, a representative of the branch organization of service users and family organizations, and researchers in the field of mental health care. During a 2-day meeting (June, 2014) the vision and mission of the model were established, the target population of the care model was defined, and core elements of the model were described. Moreover, the title of the model was determined: active recovery triad (ART). At the end of the meeting, a writing team was formed consisting of six people from the expert group in order to work out the key characteristics and the vision of the new care model into a handbook for professionals (TvM, LvdM, YV, BB, BS, and JvW).

The outcomes of the 2-day meeting were presented during a second invitational meeting (September, 2014). Sixty-seven people attended this meeting (e.g., mental health workers, policy advisors, managers, directors, researchers, peer workers, and family representatives). The goal of this meeting was to reflect upon the outline of the care model. Six topics were discussed in small groups: namely, peer support, a model fidelity scale, professional and personal values working with the ART model, enlarged passion of care workers, paradigm shift in mental health care, and what we can learn from other countries. During this meeting, the need for an increase in service user and family perspectives in the process of model development became apparent.

In order to investigate the perspectives of service users and family with regard to the ART model and secure these perspectives in the model development process, a second 2-day meeting was organized (December, 2014). This meeting commenced with a morning session in which five service user representatives or peer workers, five family representatives, and four mental health professionals from the expert group reflected upon the ongoing ART model development from their own perspectives. The conclusions of this meeting were integrated into the second 1.5-day meeting, in which the total expert group defined seven steps in care important for the ART model and created an outline for a model fidelity scale.

After the second invitational meeting, the writing team drafted the first chapters of the ART handbook. These chapters were sent to the attendants prior to a third invitational meeting that 81 people attended (April, 2015). During this meeting, the content of the first chapters of the handbook were discussed in small groups, and experiences with best practices in the field of long-term mental health care were exchanged.

The fourth and last invitational meeting took place in November, 2015. During this meeting, the draft version of the ART handbook was presented. The aim of the meeting was to collect feedback in order to finalize the handbook. Seventy-one attendants were able to indicate the topics they still missed in the handbook. The three main topics that were mentioned by the attendants were (1) provide information on available knowledge regarding recovery, (2) focus more on how to reduce loneliness among service users, and (3) concretize the added value of ART to clinical practice as well as how to implement the model in practice. In addition, workshops in small groups were organized to fine tune the content of the different chapters of the handbook. Based on this input, the writing team finished the ART handbook, which was published in June, 2016 (17).

No ethical approval was necessary, since this manuscript describes a development process in care practice rather than empirical research involving human subjects.

## Results

ART is an acronym for Active Recovery Triad. It entails an integral care model for long-term mental health care aiming at recovery for people with serious mental illness (SMI) (17). In this section, the target service user group, the ART model, and practical guidelines of the model will be described.

### Target Group

The target service user group of ART are persons of 18 years or older, diagnosed with SMI (such as bipolar disorder, mood disorder, schizophrenia, or psychotic disorder, whether or not in combination with substance abuse) and cope with serious mental and social consequences of their disorder. The impairments of people included in the target group of ART follow a chronic course and people have faced multiple unsuccessful attempts toward more independence and recovery. They are currently dependent on 24-h care and support in either a long-term clinical ward, residential facility, or supported accommodation. There are no further exclusion criteria, such as substance abuse.

### The ART Model

#### Active

The first core principle of the model is an emphasis on active engagement of all agents in the triad: care workers, service users, and significant others. Service users should be active agents in the recovery process, including their options for treatment and living accommodation (18). An anticipated timeframe for the duration of stay has been demonstrated to be positively associated with successfully leaving long-term mental health settings, presumably since this improves a goal-directed treatment and support (33). The timeframe should be long enough to work on small steps in recovery, but short enough to prevent chronic hospitalization. Therefore, consensus was reached upon a timeframe of 3 years in the art model, which was regarded as optimal by stakeholders involved in the development process. In case of insufficient recovery to move to more independent living after 3 years, an evaluation of the provided care and rehabilitation is essential to reconsider the treatment and rehabilitation plan. This evaluation should be performed with a third independent party, for example, an independent organization providing consultation and expertise, to ensure critical evaluation, and provide new insights into the possibilities of the treatment and rehabilitation plan.

#### Recovery

For service users of long-term facilities, the concept “recovery” is often unknown, or, when associated with full remission of symptoms, perceived as not feasible. Therefore, it is important to introduce the actual meaning of the concept of recovery and empower service users to pursue steps toward recovery. The expertise of peer workers to create this awareness among service users and family is crucial (36, 37). The ART model distinguishes four dimensions of recovery, namely, recovery of health, recovery of personal identity, recovery of daily functioning, and recovery of community functioning (38). Regardless of dimension, a paradigm shift is necessary toward thinking in terms of strengths and possibilities rather than in problems. This accounts for care workers, but also for service users and significant others. The first-dimension recovery of health refers to physical as well as mental health and the intertwinement between these two, including attention for lifestyle, polypharmacy, and general well-being of the service users. Important is the cooperation between professionals, such as mental health workers, general practitioners, dentists, etc. The second dimension captures recovery of identity, involving the quest of (re)discovering someone's identity and exploring their life story, which is of great importance in order to (re)gain autonomy. Knowledge and experiences of family and significant others can contribute to the life story of service users. The third dimension entails recovery of daily functioning and refers to supporting and stimulating service users as much as possible to become more self-reliant when it comes to daily tasks like cooking, cleaning, grooming, etc. Care workers should be aware not to manage common daily tasks for service users, but together search for more independence. Important is the specific attention for daily activities and a healthy day–night cycle. The last dimension is recovery of community functioning and refers to the importance of participation and obtaining a social role in society, for example, cooperation with the community in terms of existing initiatives instead of separate activities in institutional setting. In literature, several rehabilitation and psychosocial interventions are described that can contribute to these dimensions of recovery and can be introduced in the context of ART. Examples are the wellness recovery action plan [WRAP (32), cognitive adaptation training (CAT) 28, 29], illness management and recovery (IMR) (39), (peer supported) open dialogue (40), and individual placement and support (IPS) (30) [see for an overview: (34, 35)].

#### Triad

The third principle of the ART model refers to the triad of the mental health workers, the service user, and significant others (family, friends, acquaintances from the past, neighbors, etc.). When contact with family or significant others is limited or absent, care workers should support service users to identify who was important for them in the past. As the family association involved in the development of the ART model pointed out: “*almost always there is a moment in time that can be recognized as the moment the contact between service user and family was seriously damaged or abandoned altogether*.” Together with the service user, care workers should explore why this contact was disrupted and help to restore this contact, if necessary, with the help of a family peer worker. The triad should be active on three different levels within the mental health organization. First, cooperation within the triad is important on the level of the individual service user, so with regard to the therapeutic relationship. Second, the triad should be represented at the level of the team, that is, the perspective of peer workers and family peer workers should be included in the team process. Finally, on the organizational level, service users and family members should be involved in policy development and organizational change.

### Practical Guidelines and Model Fidelity Scale

In the ART handbook, the three core principles of ART are translated into practical guidelines (17). In order to structure the care process, seven steps are defined.

The first step is an intake meeting (1), where the indication criteria of ART are examined, and the personal story of the service user is explored. Before admission, it is discussed whether ART is the warranted department and if all recommended (evidence-based) treatment options have been considered. Establish and maintain contact with the service user, building a relationship of trust (2) is important from the start and a Care Planning Meeting (CPM) is organized within the first week of admission (3), in order to discuss treatment, support, and interventions in alignment with the personal goals of the service user and why previous (rehabilitation) treatments have been unsuccessful. This CPM needs to be organized every 6 months. Family and significant others should be involved in these meetings, and if contact is minimal or absent, focus should be on restoring this contact (4). The concept of recovery is unfamiliar for the majority of the service users and should be introduced and explained to service users and their significant others (5). The next step is defining the needs, strengths, and wishes of the service user (6) and the formulation of personal recovery goals. This is the basis of a treatment and rehabilitation plan (7), to structure the care, support, and recovery interventions. At least every 6 months, during the CPM, the personal recovery goals are evaluated, and new goals might be formulated, based on the needs, wishes, and recovery process of the service user.

These seven steps provide guidance to professionals on how to work with service users on the four dimensions of recovery. The steps can be considered as a practical elaboration of the basic care process, but do not necessarily need to be performed in this specific order. Additionally, care workers are free in their manner of adoption of these steps as well as the tools and methods they deem appropriate. Of course, the wishes and needs of service users should be the key driver. Recovery oriented care is the basis of care practice, which is not only visible in these practical steps but also in the contact between care workers, service users, and significant others, the attitude of care workers, the vision of the team, and the culture within the organization.

The ART model is operationalized into a model fidelity scale, describing all components important for the ART model in a quantitative way: “the ART monitor.” The instrument consists of 51 items, subdivided into nine domains: (1) team structure, (2) team process, (3) recovery-oriented care and treatment, (4) other principles of recovery-oriented care and treatment, (5) organization of care, (6) professionalization, (7) architectural design, (8) safety, and (9) legislation regarding coercion. The ART monitor can be used to measure the degree of adherence to the ART model within a team by means of audits, performed by independent auditors. Auditors can be professionals of different disciplines (e.g., peer workers, family representatives, social workers, nurses, nurse practitioners, psychiatrists, and managers) who received a training on how to conduct an audit. Based on a 1-day audit, the auditors score the ART monitor. The items of the ART monitor can be scored on a five-point Likert scale based on the degree of compliance to the ART model, ranging from 1 (not compliant) to 5 (fully compliant). Table 2 provides an overview of the nine domains accompanied by some examples of the items within the instrument.

**Table 2:** Domains of the active recovery triad (ART) monitor and examples of items.

**Domain**	**Example of items**
1. Team structure	Team composition Peer worker and family peer worker
2. Team process	Vision/working methods Hospitality and presence
3. Recovery oriented care and support	Needs, strengths, and wishes Recovery interventions on four levels
4. Other principles of recovery-oriented care and support	Somatic care Dual diagnosis
5. Organization of care	Cooperation with FACT and other outpatient care teams Care process and consultation
6. Professionalization	Reflection Team spirit
7. Healing environment	Healing environment Conditions of housing accommodations
8. Safety	Conflict control and personal safety Cooperation agreements concerning safety
9. Reduction of coercion	Evaluation of coercive measures

The ART handbook and the model fidelity scale are useful tools for professionals in order to implement the ART model in practice and can support the team in deciding which concrete steps are necessary to improve care. A large national research project took place on validating the model fidelity scale in order to ensure a valid and reliable tool to measure the degree of adherence to the ART model in a team. Currently, a manuscript of this study is in preparation.

## Discussion

The active recovery triad (ART) model is a framework for Dutch long-term mental health care, especially for the low-volume high-need group of people with SMI who are admitted for a long time (17). ART combines the focus on recovery with the notion of active cooperation in the triad of professionals, service users, and significant others. Working with the ART model requires a variety of disciplines within the team, a critical evaluation of the care and support that is available for service users, a healthy and recovery supporting living environment, and an increase in variety of treatment and (evidence-based) rehabilitation interventions. This change is needed in order to provide the group service users in long-term mental health care-tailored support, with tools to take steps in their recovery process.

The development of the ART model is in line with the recovery movement and the focus on rehabilitation for people with SMI that started in the mid-twentieth century (1–3). Especially the focus on personal recovery as an important process in addition to clinical recovery is in agreement with international literature (3, 41). In other countries, comparable developments have taken place, for example, the community care units (CCUs) in Australia, especially the Transitional Residential Rehabilitation type (42, 43). CCUs are facilities located within the community where 24-h care and support are provided by a multidisciplinary team. CCUs resemble the ART model in the aim to assist long-stay service users to more independent living, the focus on recovery, the close cooperation with resources in the community, and the focus on a temporary stay. However, literature regarding CCUs describe facilities in the community rather than an integral care model as ART, which is based on an underlying vision and core principles (42–45). The ART model can be implemented in teams operating within the community but also teams situated at large institutional grounds, to improve the situation in this setting. In addition, the ART monitor enables teams and mental health organizations to measure the degree of adherence to the ART model, whereas CCUs are not explicated in a model fidelity scale. This is important, as it provides the teams and organizations with a framework that supports them in the identification of concrete improvement areas and makes improving quality of care more feasible and within reach.

Comparable developments around recovery-oriented care took place in the UK (46). An example is the REFOCUS intervention aiming to promote personal recovery of service users (47). It includes recovery-promoting relationships, by offering training for staff on personal values, promoting knowledge, and developing coaching skills. It also focuses on understanding service users' values and treatment preferences, identifying strengths and abilities and supporting personal goals. However, the REFOCUS intervention mainly involves care practice aiming to improve personal recovery of service users, whereas the ART model has a broader aim and also sets standards on organizational and policy level, such as care organization, team structure, and housing facilities. Another established instrument in the UK, similar to the ART model, is the QuIRC developed to assess the quality of long-term mental health facilities (48). When comparing the ART model with the principles of the QuIRC, we see various similarities (48). Important parallels are the focus on a broad definition of recovery, the emphasis on the involvement of service users in decision making and policy development, a safe and homely environment, the cooperation with organizations in the community, and a certain team composition and competencies of the team. However, some differences come to the fore as well. First, in terms of the development process, the origin of the ART model lays within the mental health practice, the model was developed in close collaboration with a large group of stakeholders, and connected to this process, the model fidelity scale was established. The QuIRC was developed based on key principles of rehabilitation described in literature, instead of an underlying care model to implement in practice (26). In addition, whereas the QuiRC mentions the involvement of family, the ART model considers family and significant others as active partners in the triad. This means that they should not only be updated about the status of the treatment, but should actively be involved in decision making on the individual level, and policy development on team and organizational level. Aspects less visible in the ART model, but explicitly addressed in the QuIRC are the attention for sexual health, diets and healthy meals, physical disabilities of service users, and the adaptation of the living environment to these disabilities; these aspects might be relevant for further development of the ART model.

To conclude, the ART model is in line with comparable international developments regarding recovery-oriented care. The ART model is distinctive from other care models and interventions in its extensiveness as a care model for all aspects of care, including recovery-oriented interventions, care organization (in terms of policy as well as more practical organizational issues), and cooperation with significant others and the community. In addition, the ART model has already become widely accepted in Dutch mental health care since the publication of the handbook in 2016. Part of the acceptance of the ART model can be ascribed to the involvement of many stakeholders, thereby incorporated perspectives of mental health professionals, service users, family, and significant others. A large number of organizations throughout the country are in the process of implementing the model into practice, using practical tools the ART handbook provides and the model fidelity scale to support this implementation process. Twenty Dutch mental health organizations participated in research on the validity and reliability of the ART monitor that was conducted between 2017 and 2019. To date, the impact of ART within Dutch care practice is still expanding since more organizations start to implement the model and are also connected to the national ART research. First indications suggest that some service users take steps in their recovery process, even though care workers were initially not convinced this would be possible. However, these effects are in need of further investigation. Therefore, an effectiveness study of the ART model on quality of care, recovery, and autonomy of service users and cooperation in the triad is underway.

## Data Availability Statement

The raw data supporting the conclusions of this article will be made available by the authors, without undue reservation.

## Author Contributions

LM, JW, TM, BB, BS, and YV took the lead in developing the ART model. LZ wrote the manuscript. LM, JW, GW, and YV participated in drafting and revising the article. All authors contributed to and agreed upon the final version of the manuscript.

## Conflict of Interest

The authors declare that the research was conducted in the absence of any commercial or financial relationships that could be construed as a potential conflict of interest.

## References

[1] Holloway F Kalidindi S Killaspy H Roberts G Enabling Recovery: The Principles and Practice of Rehabilitation Psychiatry. (2015) London: The Royal College of Psychiatrists.

[2] Schrank B Slade M Recovery in psychiatry. Psychiatria Danubina. (2007) 19:246–51. 10.1192/pb.bp.106.013425

[3] Slade M Amering M Oades L Recovery an international perspective. Epidemiol Psichiatr Soc. (2008) 17:128–37. 10.1017/S1121189X00002827 18589629

[4] van Weeghel J van Zelst C Boertien D Hasson-Ohayon I. Conceptualizations, assessments, and implications of personal recovery in mental illness: a scoping review of systematic reviews and meta-analyses. Psychiatr Rehabil J. (2019) 42:169. 10.1037/prj0000356 30843721

[5] World Health Organization Mental Health Action Plan 2013-2020. (2013) Available online at: apps.who.int/iris/bitstream/10665/89966/1/9789241506021_eng.pdf.2 (accessed December 17, 2019).

[6] World Health Organization Europe The European Mental Health Action Plan 2013–2020. (2015) Available online at: https://www.euro.who.int/__data/assets/pdf_file/0020/280604/WHO-Europe-Mental-Health-Acion-Plan-2013-2020.pdf (accessed December 17, 2019).

[7] European Commission Green Paper: Improving the Mental Health of the Population: Towards a Strategy on Mental Health for the European Union. (2005). Available online at: ec.europa.eu/health/ph_determinants/life_style/mental/green_paper/mental_gp_en.pdf (accessed December 17, 2019).

[8] Killaspy H McPherson P Samele C Keet R Almeida JCd EU Compass for Mental Health and Well-being: Providing Community Based Mental Health Services (Position Paper). (2014) Available online at: pdfs.semanticscholar.org/e8e6/e07e32cfeb0589955dac5035d0a24f050b95.pdf (accessed December 17, 2019).

[9] Nederland GGZ Naar herstel en gelijkwaardig burgerschap: Visie op de langdurende zorg aan mensen met ernstige psychische aandoeningen. (2009) Available online at: www.ggznederland.nl/uploads/assets/asset_305955.pdf (accessed December 17, 2019).

[10] Projectgroep Plan van Aanpak EPA Crossing the Bridge. Kenniscentrum Phrenos. (2014) Available online at: www.kenniscentrumphrenos.nl/wp-content/uploads/2014/10/Over-de-brug-PvA-EPA-september-2014.pdf (accessed July 19, 2019).

[11] Parabiaghi A Bonetto C Ruggeri M Lasalvia A Leese M Severe and persistent mental illness: a useful definition for prioritizing community-based mental health service interventions. Soc Psych Psych Epid. (2006) 41:457–63. 10.1007/s00127-006-0048-0 16565917

[12] Delespaul PH De Consensusgroep EPA. Consensus over de definitie van mensen met een ernstige psychische aandoening (EPA) en hun aantal in Nederland. Tijdschrift voor de Psychiatrie. (2013) 55:427–38. Available online at: https://www.tijdschriftvoorpsychiatrie.nl/assets/articles/55-2013-6-artikel-delespaul.pdf 23864410

[13] Anthony WA Recovery from mental illness: the guiding vision of the mental health service system in the 1990s. Psychosoc Rehabil J. (1993) 16:11–23. 10.1037/h0095655

[14] Prinsen E van Wel B Mulder N Koning N Handboek IHT: Intensive Home Treatment. Utrecht: De Tijdstroom (2016).

[15] van Mierlo T Bovenberg F Voskes Y Mulder N Werkboek HIC: High and Intensive Care in de Psychiatrie. Utrecht: De Tijdstroom (2013).

[16] van Veldhuizen JR. FACT: a Dutch version of ACT. Community Ment Health J. (2007) 43:421–33. 10.1007/s10597-007-9089-4 17514502

[17] van Mierlo T van der Meer L Voskes Y Berkvens B Stavenuiter B van Weeghel J De kunst van ART. Werkboek Active Recovery Triad. Utrecht: De Tijdstroom (2016).

[18] Leonhardt BL Huling K Hamm JA Roe D Hasson-Ohayon I McLeod HJ. Recovery and serious mental illness: a review of current clinical and research paradigms and future directions. Expert Revi Neurother. (2017) 17:1117–30. 10.1080/14737175.2017.1378099 28885065

[19] Killaspy H. The ongoing need for local services for people with complex mental health problems. Psychiatr Bull. (2014) 38:257–9. 10.1192/pb.bp.114.048470 25505623 PMC4248159

[20] Trieman N Leff J. Long-term outcome of long-stay psychiatric in-patients considered unsuitable to live in the community: TAPS Project 44. Br J Psychiat. (2002) 181:428–32. 10.1192/bjp.181.5.428 12411270

[21] Wiersma D Nienhuis FJ Slooff CJ Giel R. Natural course of schizophrenic disorders: a 15-year followup of a Dutch incidence cohort. Schizophr Bull. (1998) 24:75–85. 10.1093/oxfordjournals.schbul.a033315 9502547

[22] Kroon H Michon H Knispel A van Erp N Hulsbosch L de Lange A Landelijke Monitor Ambulantisering en Hervorming Langdurige GGZ 2019. (2019) Available online at: https://www.trimbos.nl/docs/046adf53-3b4b-4aa0-9635-01641b301fc1.pdf (accessed July 19, 2019).

[23] Priebe S Frottier P Gaddini A Kilian R Lauber C Martínez-Leal R. Mental health care institutions in nine European countries, 2002 to 2006. Psychiatr Serv. (2008) 59:570–3. 10.1176/ps.2008.59.5.570 18451020

[24] Advies Commissie Toekomst beschermd wonen (In opdracht van de Vereniging van Nederlandse Gemeenten) Van beschermd wonen naar een beschermd thuis. Vereniging van Nederlandse Gemeenten. (2015) Available online at: https://vng.nl/files/vng/van-beschermd-wonen_20151109.pdf

[25] Barton R Institutional Neurosis. Bristol: Butterworth-Heinemann (1976).

[26] Killaspy H King M Wright C White S McCrone P Kallert T. Study protocol for the development of a European measure of best practice for people with long term mental health problems in institutional care (DEMoBinc). BMC Psychiatry. (2009) 9:36. 10.1186/1471-244X-9-36 19523240 PMC2702375

[27] Killaspy H Cardoso G White S Wright C Caldas de Almeida JM Turton P. Quality of care and its determinants in longer term mental health facilities across Europe; a cross-sectional analysis. BMC Psychiatry. (2016) 16:31. 10.1186/s12888-016-0737-5 26868834 PMC4750356

[28] Stiekema AP Quee PJ Dethmers M van den Heuvel ER Redmeijer JE Rietberg K. Effectiveness and cost-effectiveness of cognitive adaptation training as a nursing intervention in long-term residential patients with severe mental illness: study protocol for a randomized controlled trial. Trials. (2015) 16:49. 10.1186/s13063-015-0566-8 25887511 PMC4327948

[29] Velligan DI Mahurin RK Eckert SL Miller AL BowThomas CC Cognitive adaptation training: the use of compensatory strategies for inpatients and outpatients with schizophrenia. Schizophr Res. (1997) 24:229 10.1016/S0920-9964(97)82662-X

[30] van Weeghel J Bergmans C Couwenbergh C Michon H de Winter L. Individual placement and support in the Netherlands: past, present, and future directions. Psychiatr Rehabil J. (2019) 43:24–31. 10.1037/prj0000372 31204822

[31] Sanches SA Swildens WE van Busschbach JT Stant AD Feenstra TL van Weeghel J. Cost effectiveness and budgetary impact of the Boston University approach to psychiatric rehabilitation for societal participation in people with severe mental illness: a randomised controlled trial protocol. BMC Psychiatry. (2015) 15:217. 10.1186/s12888-015-0593-8 26373711 PMC4571072

[32] Cook JA Copeland ME Jonikas JA Hamilton MM Razzano LA Grey D. Results of a randomized controlled trial of mental illness self-management using wellness recovery action planning. Schizophr Bull. (2012) 38:881–91. 10.1093/schbul/sbr012 21402724 PMC3406522

[33] Taylor Salisbury T Killaspy H King M. The relationship between deinstitutionalization and quality of care in longer-term psychiatric and social care facilities in Europe: a cross-sectional study. Eur Psychiat. (2017) 42:95–102. 10.1016/j.eurpsy.2016.11.011 28364688

[34] van der Meer L Wunderink C. Contemporary approaches in mental health rehabilitation. Epidemiol Psychiatr Sci. (2019) 28:9–14. 10.1017/S2045796018000343 30043719 PMC6998946

[35] Bitter N Roeg D Van Nieuwenhuizen C van Weeghel J. Recovery in supported accommodations: a scoping review and synthesis of interventions for people with severe mental illness. Community Ment Health J. (2020) 56:1–24. 10.1007/s10597-020-00561-3 32016620 PMC7289772

[36] Mahlke CI Priebe S Heumann K Daubmann A Wegscheider K Bock T. Effectiveness of one-to-one peer support for patients with severe mental illness—a randomised controlled trial. Eur Psychiat. (2017) 42:103–10. 10.1016/j.eurpsy.2016.12.007 28364685

[37] Solomon P. Peer support/peer provided services underlying processes, benefits, and critical ingredients. Psychiatr Rehabil J. (2004) 27:392–401. 10.2975/27.2004.392.401 15222150

[38] Dröes J Plooy A Herstelondersteunende zorg in Nederland: een vergelijking met Engelstalige literatuur. Tijdschrift voor Rehabilitatie. (2010) 19:6–17. Available online at: https://www.ggzdigitaal.nl/p/herstelondersteunende-zorg-in-nederland-vergelijking-met-engelstalige-literatuur/2287

[39] Mueser KT Corrigan PW Hilton DW Tanzman B Schaub A Gingerich S. Illness management and recovery: a review of the research. Psychiatr Serv. (2002) 53:1272–84. 10.1176/appi.ps.53.10.1272 12364675

[40] Seikkula J Open dialogue integrates individual and systemic approaches in serious psychiatric crises. Smith Coll Stud Soc Work. (2003) 73:227–45. 10.1080/00377310309517683

[41] Whitley R Drake RE. Recovery: a dimensional approach. Psychiatr Serv. (2010) 61:1248–50. 10.1176/ps.2010.61.12.1248 21123410

[42] Parker S Hopkins G Siskind D Harris M McKeon G Dark F. A systematic review of service models and evidence relating to the clinically operated community-based residential mental health rehabilitation for adults with severe and persisting mental illness in Australia. BMC Psychiatry. (2019) 19:55. 10.1186/s12888-019-2019-5 30717713 PMC6360669

[43] Trauer T Farhall J Newton R Cheung P. From long-stay psychiatric hospital to Community Care Unit: evaluation at 1 year. Soc Psychiatry Psychiatr Epidemiol. (2001) 36:416–9. 10.1007/s001270170032 11766972

[44] Chopra P Harvey C Herrman H Continuing accommodation and support needs of long-term patients with severe mental illness in the era of community care. Curr Psychiatry Rev. (2011) 7:67–83. 10.2174/157340011795945801

[45] Meehan T Stedman T Parker S Curtis B Jones D. Comparing clinical and demographic characteristics of people with mental illness in hospital-and community-based residential rehabilitation units in Queensland. Aust Health Rev. (2017) 41:139–43. 10.1071/AH15207 27119964

[46] Bird V Leamy M Le Boutillier C Williams J Slade M REFOCUS: Promoting Recovery in Mental Health Services. (2014) London: Rethink Mental Illness.

[47] Slade M Bird V Le Boutillier C Farkas M Grey B Larsen J. Development of the REFOCUS intervention to increase mental health team support for personal recovery. Br J Psychiatry. (2015) 207:544–50. 10.1192/bjp.bp.114.155978 26450586

[48] Killaspy H White S Wright C Taylor TL Turton P Kallert T. Quality of longer term mental health facilities in Europe: validation of the quality indicator for rehabilitative care against service users' views. PLoS ONE. (2012) 7:e38070. 10.1371/journal.pone.0038070 22675508 PMC3366953

